# The required competencies of physicians within palliative care from the perspectives of multi-professional expert groups: a qualitative study

**DOI:** 10.1186/s12904-020-00566-5

**Published:** 2020-05-09

**Authors:** Hanna-Leena Melender, Minna Hökkä, Tiina Saarto, Juho T. Lehto

**Affiliations:** 1 Department of Social and Health Care, VAMK University of Applied Sciences, Wolffintie 27-31, 65200 Vaasa, Finland; 2 School of Health, Kajaani University of Applied Sciences, PL 52, Ketunpolku 4, 87101 Kajaani, Finland; 3 Faculty of Medicine, University of Helsinki, Helsinki, Finland; 4 Helsinki University Hospital, Cancer Center, PL 180, 00029 HUS Helsinki, Finland; 5 Faculty of Medicine and Health Technology, Tampere University, Tampere, Finland; 6 Department of Oncology, Palliative Care Unit, Tampere University Hospital, Teiskontie 35, R-building, 33520 Tampere, Finland

**Keywords:** Palliative medicine, Palliative care, Professional competence, Clinical competence, Curriculum, Education, Qualitative research

## Abstract

**Background:**

Although statements on the competencies required from physicians working within palliative care exist, these requirements have not been described within different levels of palliative care provision by multi-professional workshops, comprising representatives from working life. Therefore, the aim of this study was to describe the competencies required from physicians working within palliative care from the perspectives of multi-professional groups of representatives from working life.

**Methods:**

A qualitative approach, using a workshop method, was conducted, wherein the participating professionals and representatives of patient organizations discussed the competencies that are required in palliative care, before reaching and documenting a consensus. The data (*n* = 222) was collected at workshops held in different parts of Finland and it was analyzed using a qualitative content analysis method.

**Results:**

The description of the competencies required of every physician working within palliative care at the general level included 13 main categories and 50 subcategories in total. ‘Competence in advanced care planning and decision-making’ was the main category which was obtained from the highest number of reduced expressions from the original data (f = 125). Competence in social interactions was another strong main category (f = 107). In specialist level data, six main categories with 22 subcategories in total were found. ‘Competence in complex symptom management’ was the main category which was obtained from the biggest number of reduced expressions (f = 46). A notable association between general level and specialist level data was related to networking, since one of the general level categories was ‘Competence in consultations and networking’ (f = 34) and one of the specialist level categories was ‘Competence to offer consultative and educational support to other professionals’ (f = 30). Moreover, part of the specialist level results were subcategories which belonged to the main categories produced from the general level data.

**Conclusions:**

The competencies described in this study emphasize decision-making, social interactions and networking. It is important to listen to the voices of the working-life representatives when planning curricula. Moreover, the views of the working-life representatives inform how the competencies gained during their education meet the challenges of the ordinary work.

## Background

About 20 million people worldwide are annually in need of palliative care and this demand is increasing in Europe due to the ageing population and the high prevalence of noncommunicable diseases [1]. The Assembly from the Council of Europe calls on Member States to strengthen palliative-care services and to ensure the adequate training of palliative care for health-care professionals [2, 3].

Palliative care services can be categorized into a minimum of two or three levels. They are named as the palliative care approach, general palliative care and specialist palliative care [4, 5]; or primary, secondary and tertiary palliative care [6]. To provide quality palliative care, health-care professionals of all levels should have sufficient competencies in palliative care [7, 8].

The concept of ‘competence in medicine’ may be defined as a holistic combination of the knowledge, skills, values or attitudes required for the effective performance of specified activities [9]. In the White Papers published by the European Association for Palliative Care (EAPC), important competencies for clinical practice in palliative care are presented for all practitioners, with 10 core interdisciplinary competencies [4, 10]. The EAPC has also presented recommendations for the development of undergraduate curricula in palliative medicine at European medical schools, and many taskforces have conducted surveys on the education in palliative medicine for physicians in Europe [11]. In Ireland, the Palliative Care Competence Framework was published in 2014 [5]. In a subsequent survey [12], mainly positive results were found when assessing the knowledge, attitude and behavior of the key competencies of physicians. However, there was also some variation in the scores.

In Finland, a national quality criterion complying with the recommendations of the EAPC [7, 8] defines that within the specialized level of palliative care, the personnel should have a special education of palliative care and work as a multi-professional expert team, while the undergraduate education for health care professionals should provide the ground level of competency to work within the basic level of palliative care [13]. Health-care units providing a specialized level of palliative care include palliative care units in hospitals, hospices and palliative home-care units (hospitals at home). All other units, such as ‘ordinary’ hospital wards or nursing homes, provide a general level of palliative care [14]. In this study, the expression ‘specialized level competencies’ refers to competencies needed when providing palliative care in specialized level units as defined here. The expression ‘general level competence’ refers to competencies needed by all physicians when providing basic palliative care, for example, at ‘ordinary’ hospital wards or nursing homes.

Education in palliative care varies in Finland. Out of the five faculties of medicine, only two have a curriculum and chair in palliative medicine. No postgraduate education in palliative care for physicians is available at the universities, although special competence in palliative medicine, awarded by the Finnish Medical Association, has been available since 2007. To meet these educational challenges, the EduPal project (Developing Palliative Nursing and Medical Education through Multidisciplinary Cooperation and Working-life Collaboration), funded by the Ministry of Education and Culture in Finland, aims to develop national recommendations for both undergraduate and specialist (postgraduate) education in palliative medicine, among other areas. As a part of the project, the required competencies of professionals working within palliative care at the general and specialist levels, described above in a Finnish context, were explored in multi-professional workshops.

## Methods

The aim of this study was to describe the required competencies of physicians working within different levels of palliative care, from the perspectives of multi-professional groups of representatives from working life.

The study is a descriptive qualitative research design with written material provided by multi-professional groups working in workshops arranged for the purpose of the study.

### Data collection and sample

The data was collected in workshops attended by a purposive sample of professionals working within the field of palliative care, in order to present a diversity of health care organizations, as well as representatives of patient organizations (Table 1). To find the best informants, managers were contacted and asked to propose the best representatives of their personnel to describe the required competencies of professionals in palliative care. Based on the managers’ recommendations, an invitation letter was sent to the persons proposed. The letter included information about the purpose of the study purpose, the reasons for the study and the persons responsible for it.

**Table 1:** Professionals who participated in workshops

Profession	Number of professionals
Physician on general level of palliative care	12
Physician on specialist level of palliative care	16
Registered nurse on general level of palliative care	63
Registered nurse on specialist level of palliative care	69
Licenced practical nurse on general level of palliative care	25
Licenced practical nurse on specialist level of palliative care	10
Expert of a third sector organization	7
Elderly care professional	1
Social worker	3
Physiotherapist	3
Nursing manager	9
Spiritual care professionals	4
**Total**	**222**

A descriptive qualitative approach was adopted. No particular disciplinary or methodological roots are claimed. The intention is to simply present a comprehensive summary of the phenomenon of interest [15]. The features of the research group members are presented in Table 2.

**Table 2:** Features of the research team members

Author (gender)	Credentials Occupation at the time of the study	Education on qualitative research methods	Experience on qualitative research methods
H-L.M. (female)	RNM, PhD, Docent Principal Lecturer at a University of Applied Science Docent in a University	Formal Master and PhD level courses on qualitative research methods	Has used qualitative methods in research work earlier. Has teaching and thesis supervisor experience on qualitative research methods.
M.H. (female)	RN (Master), MNSc, PhD-student Senior Lecturer and Project Manager at a University of Applied Science	Formal Master and PhD level courses on qualitative research methods	Has used qualitative methods in research work earlier. Has teaching and thesis supervisor experience on qualitative research methods.
T.S. (female)	MD, PhD Professor in a University and Chief Physician in a University Hospital	Informal learning activities to embrace the principles of qualitative research methods	Has used qualitative methods in research work earlier. Has thesis supervisor experience on qualitative research methods.
J.L. (male)	MD, PhD, Docent Clinical teacher in a University and Chief Physician in a University Hospital	Informal learning activities to embrace the principles of qualitative research methods	Has used qualitative methods in research work earlier.

The 21 workshops were organized by the teachers of the participating universities of applied sciences (UAS) and they took place either at the UASs or at the workplaces of the workshop members. Some workshops covered more than one working group (WG), because in some cases, a general level group and a specialist level group were invited to the same workshop. All in all, data was obtained from 36 WGs in 21 workshops. The teachers of the UASs acted as moderators. Of the research team members, one (H-L.M.) acted as a moderator in one workshop. The participants were informed that all the moderators had an interest in developing palliative care through the development of the professionals’ education. The moderators did not establish any relationship with the participants prior to commencement of the study. In addition to the participants and the moderators, no other persons were present in the workshops.

The workshops started with a presentation about the project and instructions for the workshop activities. The WGs received a questionnaire which had been developed for the purposes of this project. This included 10 open-ended questions regarding the required competencies of health care professionals and other aspects concerning the development of palliative care. The questionnaire had been pre-tested among one WG. Based on the pre-test, no changes had been made to the questionnaire and the pre-test data was included in the research data. The workshop members worked through the questionnaire, discussing their views on the topics with the other group members and documenting their answers, mostly on paper and in some cases with a computer. The moderators were available for the purpose of clarifying any questions, but they did not participate in the discussions. However, they did observe the discussions and made field notes. The duration of the workshops varied between 2 and 4 h.

This paper reports on the findings of the data retrieved from the following questions on the physicians’ competencies in palliative care:

1. What are the required competencies of every physician in palliative care at the general level?
2. What are the required competencies of a specialized physician in palliative care at the specialist level?

### Data analysis

The original workshop data was transcribed verbatim and placed into a matrix which was presented in a Word document. In the analysis of the material, a qualitative content analysis method [16] was used. The data analysis was performed manually, i.e. using no software.

For the general level data, an inductive approach for the analysis was used, meaning that the categories emerged from the data and no theoretical framework was used in the analysis [16]. In the analysis of the specialist level data, deductive and inductive approaches fluctuated: the first part of this data was first analyzed using a deductive approach, followed by an inductive approach. The second part of the data was analyzed inductively. (Table 3.)

**Table 3:** Approaches used in the analysis of different datasets

Data	Approach
1. The general level data (all)	Inductive approach: no theoretical framework; the categories emerged from the data (Table 5)
2.The specialist level data (first part)	Deductive approach: the categorization of the general level competencies was used as a framework of the analysis (Table 6)
3.The specialist level data (second part)	Inductive approach: no theoretical framework; the categories emerged from the data which did not fit into the framework of the general level categorization (Table 6)

#### Analysis of the general level data

The inductive content analysis of the general level data was performed in three phases: reducing, clustering and abstracting. Words, phrases, sentences or units of meaning containing more than one sentence were used as units of analysis. In the reduction phase, the coding of the meaningful expressions was guided by the research question [16]. The material was read through and following questions were asked: “What are the required competencies of every physician in palliative care at the general level?” and “What are the required competencies of a specialized physician in palliative care at the specialist level?”. The codes were such expressions which gave answers to the questions. Coding was conducted manually in the matrix by coloring the codes. The codes were also restored for the clustering phase by copying and pasting them into a new cell of the matrix. An example of the coding process is provided in Table 4.

**Table 4:** An example of the coding procedure: how the subcategory ‘Methods of pain management’ was produced inductively

Examples of the substantive material	Reduced expressions (codes)	Subcategory
management of cancer pain catastrophizing (WG 1)	management of cancer pain catastrophizing	Methods of pain management
morphine-based pain medication (WG 2)	morphine-based pain medication	
basic morphine pain pump (WG 2)	basic morphine pain pump	
pain pump* (WG 3)	pain pump	
to be able to manage pain symptoms (WG 6))	manage pain symptoms	
Non-pharmacological pain management (WG 12)	Non-pharmacological pain management	
Physician’s sufficient medical competence when it comes to symptom management: pain medication (WG 14)	pain medication	
Physicians sufficient medical competence, for example, knowing how to prescribe the pain medication and having courage to do that. (WG 14)	prescribe the pain medication... having courage to do that	
Medication: few opioids (no fear of addiction, dosing, adverse effects, change from p.o. to s.c. etc.) (WG 15)	Medication: few opioids (no fear of addiction, dosing, adverse effects, change from p.o. to s.c. etc.)	
Starting the use of a pain pump (WG 21)	the use of a pain pump	
Competence in pain management. Basic methods, for example, pain pump – the physician has to know it (WG 22)	pain management. Basic methods … pain pump	

*) In Finland, ‘pain pump’ is a commonly used expression for equipment for patient-controlled analgesia

In the clustering phase, the codes were grouped together based on the similarity of the content and then finally, the clusters were abstracted. Abstracting meant that the clusters were shaped into sub-categories and main categories which were named based on their contents [16] (Table 5). Only the manifest content was analyzed, which means that only “what had been written” was analyzed and no interpretation about the latent intentions of the participants, for example, was done [17]. The phases were not entirely separate [17], since already during the reduction phase, clustering and abstracting started to take shape. One researcher (H-L.M.) coded and categorized the material. After that, two other members of the research group (M.H. and J.L.) studied the material and critically checked the analysis. The contents of the categories were specified together [18]. The frequencies (f) of the codes (reduced expressions) which constituted each category were counted to show the noteworthiness of the category in relation to the entirety. The number of codes in total was 573 for the general level data and 150 for the specialized level data; in both cases the data was saturated. Saturation can be defined as the point in coding where the researcher finds that no new codes occur in the data. This can be seen as a point where no new data would be needed [19]. In this study, the entire data of the 36 WGs was collected before the analysis started. During the analysis of the general level data, saturation was noticed during the coding and sorting of the codes of the WG 34 data when the same codes started to appear in the data and no new codes emerged to create any new categories in the subsequent coding. For the specialized level data, a similar notification of data saturation occurred during the coding and sorting of the codes of the WG 33 data. However, a decision was made to analyze all the data and not close the analysis at the saturation point, since we wanted to use all the valuable material that the participating WG members had provided in order to ensure that everyone’s voices would be heard.

**Table 5:** Required competencies for the general level

Main categories	Subcategories
(1.) Competence in advanced care planning and decision-making (f = 125)	(1.) Withholding therapies and setting goals of care (f = 62) (2.) Timely decision-making (f = 38) (3.) Advanced care planning (f = 19) (4.) Coordination of care (f = 6)
(2.) Competence in social interactions (f = 107)	(5.) Encountering patients and significant others (f = 37) (6.) Verbal communication (f = 16) (7.) Social interactions as part of a physician’s work in palliative care (f = 15) ** (8.) Sensitivity and empathy (f = 15) (9.) Breaking the bad news (f = 11) (10.) Professional behaviour (f = 6) (11.) Social interactions with special groups (f = 5) (12.) Active role in social interactions (f = 2)
(3.) Competence in basics of palliative care (f = 79)	(13.) Holistic attention of patient’s physical, psychosocial and existential needs (f = 22) (14.) Involvement of the significant others with care (f = 12) (15.) Recognition of the need for palliative care and practicing palliative care based on the guidelines (f = 10) (16.) Knowledge on basic principles of palliative care (f = 9) (17.) Recognition of the dying patient (f = 9) (18.) Definitions of palliative and end-of-life care (f = 8) (19.) Palliative care in different diseases (f = 5) (20.) Practices related to patient’s death (f = 4)
(4.) Competence in the management of other symptoms than pain (f = 74)	(21.) Methods of management of different symptoms (f = 45) (22.) Recognition of symptoms (f = 13) (23.) Symptom management as part of a physician’s work within palliative care (f = 11) ** (24.) Evaluation of the patient’s drug therapy within palliative care (f = 5)
(5.) Competence in consultations and networking (f = 34)	(25.) Recognition of the need for a consultation (f = 17) (26.) Skills in networking (f = 11) (27.) Consultations in a physician’s work within palliative care (f = 6)
(6.) Competence in pain management (f = 31)	(28.) Management of pain as part of a physician’s work within palliative care (f = 14) ** (29.) Methods of pain management (f = 11) (30.) Assessment of pain (f = 6)
(7.) Juridical and ethical competence (f = 30)	(31.) Respect of patient’s rights (f = 13) (32.) Patient’s autonomy (f = 6) (33.) Respect of a human being (f = 4) (34.) Honesty (f = 3) (35.) Doing good (f = 2) (36.) Patient’s freedom of choice (f = 1) (37.) Accountability (f = 1)
(8.) Patient education competence (f = 26)	(38.) Guidance of a patient and significant others as part of a physician’s work in palliative care (f = 19) ** (39.) Conduct of guidance (f = 7)
(9.) Competence in multidisciplinary teamwork (f = 21)	*- (No subcategories)*
(10.) Competence in documentation (f = 18)	(40.) Documentation of goals and limits of care (f = 9) (41.) Documentation as part of a physician’s work within palliative care (f = 3) ** (42.) Making medical certifications and verdicts (f = 3) (43.) Detailed and real time documentation (f = 2) (44.) Responding to notes (f = 1)
(11.) Competence at existential dimension (f = 12)	(45.) Relieving existential suffering (f = 7) (46.) Encountering death (f = 5)
(12.) Cultural competence (f = 10)	(47.) Significance of a cultural perspective within palliative care (f = 8) (48.) A member from another culture in a team (f = 2)
(13.) Competence in taking care of one’s own professional competence and well-being at work (f = 6)	(49.) Taking care of one’s own professional competence (f = 3) (50.) Taking care of one’s own well-being at work (f = 3)

**) Subcategories number 7, 23, 28, 38 and 41 constituted from very short and simple expressions about the thing named in the beginning of the subcategory’s name. Thus, the analysers concluded that the experts just expressed the importance of the issue within palliative care

#### Analysis of the first part of the specialist level data

The specialist level data was reduced with the same principles as the general level data described above. In a deductive approach, a structured or unconstrained matrix of analysis is operationalized based on previous knowledge such as a model or theory [16]. The choice to use this approach when analyzing the first part of the specialist level data was based on the fact that many WGs expressed in their answers that the specialist level physicians should have all the same competencies as the general level physicians, and moreover, under the same main categories, they should have some advanced competencies associated with the main categories. Based on this, the categorization of the general level competencies was used as a framework to build a structured matrix for the analysis of the first part of the specialist level data. At first, this data was coded for correspondence with the general level main categories of the framework. After that, only codes including new information, which was unique to the specialist level, were chosen for further analysis. Codes including information which had already been found in the general level data were not chosen to the specialist level categorization. The new codes were then grouped together on the basis of the similarity of the content and abstracted inductively into new sub-categories which belonged to the main categories which had already been created at the general level data analysis (Table 6).

**Table 6:** Required competencies for the specialist level

***Main categories (1, 2, 3 and 6 produced already for the general level) (see*** Table 5***)***	***Subcategories*** (Subcategories documented with Bold were created from the specialist level data)
(1.) Competence in advanced care planning and decision-making *(f = 125 on the general level)* ***(f = 126 on the general and specialist levels in total)***	*Four subcategories on the general level (see* Table 5*)* **(51.) Demanding decision-making (f = 1) (a specialist level subcategory)**
(2.) Competence in social interactions *(f = 107 on the general level)* ***(f = 110 on the general and specialist levels in total)***	*Eight subcategories on the general level (see* Table 5*)* **(52.) Special skills in social interactions (f = 3) (a specialist level subcategory)**
(3.) Competence in basics of palliative care *(f = 79 on the general level)* ***(f = 81 on the general and specialist levels in total)***	*Eight subcategories on the general level (see* Table 5*)* **(53.) Children as significant others (f = 2) (a specialist level subcategory)**
(6.) Competence in pain management *(f = 31 on the general level)* ***(f = 40 on the general and specialist levels in total)***	*Three subcategories on the general level (see* Table 5*)* **(54.) Special methods and techniques of pain management (f = 9) (a specialist level subcategory)**
**Main categories (Inductively produced for the Specialist level only)**	**Subcategories (Specialist level only)**
(14.) Competence in complex symptom management (f = 46)	(55.) Widespread and specialized symptom management as part of advanced competencies (f = 16) *** (56.) Evidence based management of symptoms (f = 14) (57.) Therapeutic procedures within palliative care (f = 10) (58.) Management of emergencies within palliative care (f = 5) (59.) Making home visits (f = 1)
(15.) Research and development competence (f = 31)	(60.) Developing palliative care (f = 23) (61.) Performing research (f = 4) (62.) Coordination of palliative care pathway (f = 4)
(16.) Competence to offer consultative and educational support to other professionals (f = 30)	(63.) Offering and coordinating consultations (f = 18) (64.) Offering education to other professionals (f = 12)
(17.) Competence to offer palliative care to all patients, including special groups (f = 14)	(65.) Children and adolescents in palliative care (f = 6) (66.) Patients with substance abuse in palliative care (f = 1) (67.) Mentally handicapped patients in palliative care (f = 1) (68.) Psychiatric patients in palliative care (f = 1) (69.) Spinal cord injury patients in palliative care (f = 1) (70.) Patients with respiratory diseases in palliative care (f = 1) (71.) Patients with heart diseases in palliative care (f = 1) (72.) Special aspects of palliative care in cancer (f = 1) (73.) Patients with rare diseases in palliative care (f = 1)
(18.) Verifiable competence to work on a specialized level of palliative care (f = 12)	(74.) Formally acquired educational competence to work on a specialized level of palliative care (f = 8) (75.) Adequate working experience needed for specialized level of palliative care (f = 4)
(19.) Competence in providing specialist level of psychosocial support (f = 2)	*- (No subcategories)*

***) Subcategory number 55 constituted from very short and simple expressions, such as “widespread symptom management” or “specialized symptom management”. Thus, the analysers concluded that the experts just expressed that specialist level physicians should have broad competence in symptom management

#### Analysis of the second part of the specialist level data

The second part of the specialist level data consisted of codes which were so unique for the specialist level physician work and differed so much from the general level data that they did not fit into the framework of the general level categorization. These codes were categorized inductively in the same way as for the general level data, by producing new main categories including relevant sub-categories (Table 6).

## Results

### The required competencies at the general level of palliative care

The description of the required competencies of every physician in palliative care at the general level included 13 main categories with a total of 50 subcategories (Table 5).

‘Competence in advanced care planning and decision-making’ was the main category from the biggest number of codes (f = 125). Examples of the original data are given below:

*“Clear instructions about the future and medication (what shall we do when the nausea increases, what shall we do when the pain increases, what shall we do if the medication does not help anymore) so that we could then react rapidly, when the situations change or the symptoms change or increase.”* (WG 7)

*“Having enough courage to make a decision about the transfer to palliative care/end-of life care.”* (WG 18).

*“Withholding therapies and making care decisions. Making decisions about end-of-life care timely. Knowing basic things about symptom management, advanced care planning”* (WG 20).

‘Competence in social interactions’ was another strong main category, as it was found in 107 codes. The following citations are examples of the original data:

“*… to be able to bring up the death coming close.”* (WG 15).

*“When it comes to psychologically and emotionally difficult decisions about care and policy, the physician has to be able to understand the need of an unhurried discussion with the patient and the family … An ability to take up the position of the patient when explaining difficult things with as ordinary spoken language as possible (not with medical terms) … Respectful encounter.”* (WG 27).

*“The art of listening and discretion.”* (WG 30).

*“Knowing how to react to the shame of the patient.”* (WG 36).

### The required competencies at the specialist level of palliative care

The first part of the specialist level results, which are subcategories belonging to the main categories produced from the general level data, are shown in the first part of Table 6. Out of them, the biggest number of codes fell into the subcategory ‘Special methods and techniques of pain management’.

As some WGs expressed:

*“Mastery of invasive pain management methods.”* (WG 12).

*“To be able to see what’s behind the pain – if there is mental agony or anxiety in the background.”* (WG 6).

The second part of the specialist level results are six main categories with 22 subcategories in total, which are unique for the specialized level only (the second part of Table 6). ‘Competence in complex symptom management’ was the main category which was derived from the biggest number of codes (f = 46). As some workgroups expressed:

“*… to be capable of providing symptom management; pleural paracentesis, sedation (for example, in psychological restlessness or anxiety). And have the courage to make the decision of sedation. The physician must be capable to see if there is emotional agony or anxiety behind the pain.”* (WG 6).

*“Management of catastrophic situations.”* (WG 12).

‘Research and development competence’ was obtained from 31 expressions. The following citations are examples of the original data:

*“Readiness to participate in research projects and development work.”* (WG 19).

*“Readiness to conduct research in the contexts of palliative care and end-of-life care”* (WG 26).

## Discussion

This study aimed to describe the required competencies of physicians in palliative care from the perspectives of multi-professional groups of representatives from working life. As a result, a comprehensive description of the required competencies based on the workshop data was presented. Earlier competence descriptions [4, 5, 10] or recommendations for the development of undergraduate curricula within palliative medicine at European medical schools, as well as surveys on the postgraduate education within palliative medicine for physicians [11], have reported some similar competencies that were also described as main categories in this study.

### General level competence needs

Within the general level, ‘Competence in advanced care planning and decision-making’ was the main category emerging with the highest number of reduced expressions. In the consensus papers by EAPC, the core competencies in palliative care include the expressions ‘tailored plan of care’, ‘decision-making’ and ‘care co-ordination’ [4, 10]. However, advanced care planning (ACP) is not mentioned as clearly as it was in this study, which emphasizes the need for well-timed decision-making and ACP as basic skills for every physician to enable high-quality palliative care. Likewise, the Irish Palliative Care Competence Framework states that as a health care professional, all physicians should demonstrate an understanding of ACP, and when they receive more training, the requirements for their competence increase to achieve skills in facilitating and leading ACP [5]. Development in medicine has increased the possibilities of taking care of patients with very advanced diseases but attempts to prolong life at any cost may be futile [20]. Recognizing the need for palliative care plays a key role in good quality care. A recent qualitative study on European experts by Paal et al. [21] reported an ability ‘to design care plans based on patients and families wishes integrating multiprofessional and interdisciplinary approaches’ as one key learning goal of postgraduate palliative care education for all healthcare providers in Europe. Thus, the WGs’ views in this study that skills in ACP are highly relevant already within the general level of palliative care are in line with previous studies and recommendations.

‘Competence in social interactions’ was another strong main category, including many diverse subcategories describing the multifaceted nature of this competence area. ‘Verbal communication’ was one of the subcategories, including similar things that were found by Paal et al. [21] who reported a learning goal ‘to listen and self-reflect’. This competence has been presented already in earlier papers [3, 10, 11]. In particular, communication skills have long been recognized as a major competency needed in palliative care [5, 11]. Today, shared decision-making with patients, families and physicians is considered the preferred model when it comes to making complex clinical decisions [22, 23]. The practice of ACP and making truly shared decisions call for skilful communication. Therefore, it is understandable why our participants emphasized competencies both in decision-making and social interactions, e.g. verbal communication. Social interactions also included aspects of teamwork, although this also had its own main category (multidisciplinary teamwork). Thus, teamwork probably had a stronger overall importance than could be stated purely by the expressions clearly related to it.

‘Competence in documentation’ was partly related to the care plan and partly related to other aspects of the physician’s work. Our result seems to highlight the importance of a written plan and decisions (e.g. DNR-orders) allowing the continuum of care between different care providers. Documentation has also been presented as a recommended learning content by EAPC [11].

Competencies in symptom control (management of pain and other symptoms) were expectedly important in basic level competencies, but the frequency of the codes related to these competencies were lower than those of decision-making and social interactions. This is not quite in line with the EAPC recommendations for undergraduate education in palliative care, where management of symptoms has the largest proportion of the total teaching time [11]. In addition, the Irish Palliative Care Competence Framework states that all physicians should understand how the palliative care approach can enhance the assessment and management of symptoms [5]. We suggest, however, that our result may reflect the significant problem in decision-making and planning palliative care as well as social interactions in Finland, rather than diminish the importance of symptom control.

### Specialist level competence needs

As for specialist level competencies, in addition to the competencies which are required on the general level, the WGs described specialized competencies which are required on this level only. ‘Competence in complex symptom management’, reported also in other papers [5, 12, 21], comprised the biggest number of reduced expressions. Although some of the expressions did not specify complex symptom management, expressions that did emerge were therapeutic procedures, taking care of emergencies and especially, the evidence base of the management. The latter is in line with the ‘Research and development competence’. This may reflect poor research activity in the field of palliative medicine, not only in Finland but worldwide. For the future development of palliative care, however, research and improvement in the academic position of palliative medicine are vital. Evidence-based practice, research and development are presented also as indicators of competence in the Irish Palliative Care Competence Framework [5].

Participants’ statements to require ‘Verifiable competence to work on a specialized level of palliative care’ are probably related to the current state of specialization within palliative medicine in Finland. There has been a special competency in palliative medicine since 2007 in Finland. This training, including 150 h of theoretical studies and a 6 months’ working period in a specialized palliative care unit, is arranged by the Finnish Association for Palliative Medicine and the Finnish Medical Association gives a certification for this special competency [24]. However, universities are not responsible for or formally involved in this education. Thus, palliative medicine is not a specialty in Finland as it is in some other European countries [25]. This might have influenced the participants’ needs to emphasize the formal special education in the specialized level of palliative care.

### Competence needs within both the general and specialist level

Consultations and networking emerged as categories ‘Competence in consultations and networking’ within the general level of palliative care and ‘Competence to offer consultative and educational support to other professionals’ within the specialized level. Consultation has been presented as an indicator of competence of all physicians in palliative care in the Irish Palliative Care Competence Framework [5]. Moreover, similar things have been presented, for example, as ‘to act as a resource to others in the team’ [10]. However, ‘Networking’ as a concept has not been presented before. Similar expressions have been presented earlier, for example, ‘fostering greater communication within the team and with other professional colleagues’ [10] and ‘establish collegial partnerships and in the context of palliative care contribute to the professional development of students, peers, colleagues and others through consultation, education, leadership, mentorship and coaching’ [5]. One plausible explanation for these results, related to consultations and networking in this study could be due to the relatively poor palliative care service network in Finland so far. This, in combination with a poor education level in this area, increases the need for consultation.

In our study, the need for organizing palliative care pathways and networks, both within the general and specialized level, emerged. This was in addition to shared decision-making and consultation. It is possible that our participants were already aware of the recently published recommendations by the Ministry of Social Affairs and Health [14] regarding palliative care, and this may have partly affected their perception of the need for palliative care in Finland.

### Strengths and limitations of the study

The trustworthiness [18] of this study was strengthened by the method being suitable for the purposes of the study. As the sample was large and presented diverse professions, it can be estimated that the data represents the whole phenomenon of interest quite well. Because the managers proposed the best representatives of their personnel, we do not know if there were refusals. None of the professionals whose contact details we received refused to participate when we contacted them. The workshop questionnaire was carefully designed and pre-tested, which strengthens the trustworthiness. The selected unit of analysis was appropriate for the purposes of this study, because it was neither too narrow nor too broad [18]. The results clearly represent the views of the participants of this study, since we analyzed only the manifest content [16].

Because the workshops were organized only once, there was no possibility to ask any further questions about the topic in order to gain a deeper understanding of the phenomenon of interest. The transcripts were not returned to the participants for comments and/or correction, nor were the findings returned so that they could provide feedback. These aspects all weaken the trustworthiness of the study. Although the sample presented diverse professions, a limitation is that the group of nurses in total was so much bigger compared with other professions and thus, the results may reflect their views more than it would reflect the others’ views. Within the Finnish health care sector, the managers are responsible for the human resource management of physicians [26] and nurses [27]. Based on this, they are supposed to know the competencies of their personnel and thus, it can be assumed that the managers succeeded at choosing the best representatives of their units for the workshops. However, it could be possible that some good experts have not been included in the sample.

One experienced qualitative researcher analyzed the whole data. Two other researchers examined the analysis made by her. The researchers exchanged views on the analysis and interpretation of the findings to create categories in a meaningful way [18]. All the authors checked that there would not be any overlap in the categorization and that it was logical. The results were presented for a multidisciplinary group of professionals within palliative care (*n* = 44). This group confirmed that the results were plausible, which confirmed the face validity of the findings. Consolidated criteria for reporting qualitative research (COREQ) guidelines [28] were adhered to ensure explicit and comprehensive reporting of the study.

At both the general and specialist level, some subcategories consisted of a small number of codes. Although these subcategories do not appear as central as the stronger ones, they are, however, important, as they show the rich and diverse nature of the competencies required in palliative care.

## Conclusions

This study adds to the knowledge by describing the perspective of Finnish multi-professional groups of representatives from working life who qualitatively described the required competencies of physicians working within different levels of palliative care. The competencies described in this study emphasize decision-making, social interactions and networking. Symptom management, which is often emphasized in curricula, also appeared in this study, but with a smaller emphasis. This may have been due to the informants considering symptom management so self-evident that they did not name it, or because the lack of competence in it may not be such a big problem in working-life, compared with the lack of other competences mentioned above. It is important to listen to the voices of the working-life representatives when planning curricula. The views of them inform how the competences gained during education meet the challenges of the ordinary work.

## Supplementary information

**Additional file 1.**

## Data Availability

The datasets generated during and/or analyzed during the current study are not publicly available due to the reassurance to the study participants that the data will be retained confidentially Within the limits of confidentiality, more detailed, but anonymous, data is available from the corresponding author on reasonable request. The English language version of the cover letter and the questionnaire developed specifically for use in this study are presented in the Supplementary material.

## Abbreviations

ACP: Advanced care planning
COREQ: Consolidated criteria for reporting qualitative research
EAPC: European Association for Palliative Care
WG: Working group
