# Supplementary material for: The required competencies of physicians within palliative care from the perspectives of multi-professional expert groups: a qualitative study
Source: BMC Palliat Care. 2020 May 9;19:65. doi: 10.1186/s12904-020-00566-5 (PMC7211329; doi:10.1186/s12904-020-00566-5)
Supplement: Supplementary file 1 — Additional file 1. [file 12904_2020_566_MOESM1_ESM.pdf]

©Hökkä, Saarto & Lehto 2018

Cover letter

Dear representative of working life,

As a part of the ’Development of Palliative Education – EduPal’ project, funded by Ministry of Education and Culture, an evaluation on competence in palliative care by the working life representatives will be made. The aim in this evaluation is to achieve a description of what competences are needed when providing palliative care at the general (basic) level (A) and specialist levels (B/C) of palliative care. Based on the descriptions gained in the first evaluation, a competence description of palliative care (physician, registered nurses, licensed practical nurses) will be made and palliative care education will be developed nationally in Finland.

You group will answer this survey anonymously and participation is voluntary. The data of this survey will be treated confidentially. The data will not be disclosed outside the research team, and individual respondents cannot be identified in the reporting of the results.

We kindly ask you, together with your group, to participate in this survey. The information you produce is important in the nationwide development of palliative care education and competencies.

Thank you.

Sincerely yours,

Minna Hökkä	Juho Lehto	Tiina Saarto
Senior lecturer	Clinical teacher	Professor
Palliative nursing	Palliative medicine	Palliative medicine
Kajaani UAS	Tampere University	Helsinki University
Tel.044-7157078	Tel. 050-4090974	Tel. 050-4270256
@:minna.hokka@kamk.fi	@:juho.lehto@staff.uta.fi	@:tiina.saarto@hus.fi

A questionnaire for health care professionals on competencies of palliative and end-of-life care

1. **The number of your group participants is _________.**
2. **What professional groups are represented in your group?**
   1. Physician_______ 2. Registered nurse________ 3. Licensed practical nurse________
   4. Other, please indicate the profession(s) ____
3. **What are the working units of your group members**? _____________________________
   ____
4. **What are the required competencies of every physician in palliative care at the general (basic) level (A)?**
5. **What are the required competencies of every registered nurse in palliative care at the general (basic) level (A)?**
6. **What are the required competencies of every licensed practical nurse in palliative care at the general (basic) level A?**
7. **What are the required competencies of every specialized physician in palliative care at the specialist levels (B/C)?**
8. **What are the required competencies of every specialized registered nurse in palliative care at the specialist levels (B/C)?**
9. **How should continuous education be provided at general (basic) level (A) to ensure the continuous development of competencies?**
10. **How should continuous education be provided at the specialist levels (B/C) to ensure the continuous development of competencies?**
11. **How do you see the appearing changes in competence needs of palliative and end-of - life care in the next ten years?**
12. **What do you see as the most important development areas in palliative and end-of-life care in the next ten years?**
13. **What do you see as the most important research areas in palliative and end-of-life care in the next ten years?**

Many thanks for taking the time and effort in answering these questions!
